# High-Sensitivity Low-Noise Miniature Fluxgate Magnetometers Using a Flip Chip Conceptual Design

**DOI:** 10.3390/s140813815

**Published:** 2014-07-30

**Authors:** Chih-Cheng Lu, Jeff Huang, Po-Kai Chiu, Shih-Liang Chiu, Jen-Tzong Jeng

**Affiliations:** 1 Institute of Mechatronic Engineering, National Taipei University of Technology, Taipei 10608, Taiwan; E-Mails: ffej918@hotmail.com (J.H.); james99918@yahoo.com.tw (P.-K.C.); aassball78@hotmail.com (S.-L.C.); 2 Department of Mechanical Engineering, National Taipei University of Technology, Taipei 10608, Taiwan; 3 Department of Mechanical Engineering, National Kaohsiung University of Applied Sciences, Kaohsiung 80778, Taiwan; E-Mail: jtjeng@kuas.edu.tw

**Keywords:** miniature fluxgate magnetometer, the second-harmonic detection, device design and simulation, flip chip, CMOS-MEMS

## Abstract

This paper presents a novel class of miniature fluxgate magnetometers fabricated on a print circuit board (PCB) substrate and electrically connected to each other similar to the current “flip chip” concept in semiconductor package. This sensor is soldered together by reversely flipping a 5 cm × 3 cm PCB substrate to the other identical one which includes dual magnetic cores, planar pick-up coils, and 3-D excitation coils constructed by planar Cu interconnections patterned on PCB substrates. Principles and analysis of the fluxgate sensor are introduced first, and followed by FEA electromagnetic modeling and simulation for the proposed sensor. Comprehensive characteristic experiments of the miniature fluxgate device exhibit favorable results in terms of sensitivity (or “responsivity” for magnetometers) and field noise spectrum. The sensor is driven and characterized by employing the improved second-harmonic detection technique that enables linear *V-B* correlation and responsivity verification. In addition, the double magnitude of responsivity measured under very low frequency (1 Hz) magnetic fields is experimentally demonstrated. As a result, the maximum responsivity of 593 V/T occurs at 50 kHz of excitation frequency with the second harmonic wave of excitation; however, the minimum magnetic field noise is found to be 0.05 nT/Hz^1/2^ at 1 Hz under the same excitation. In comparison with other miniature planar fluxgates published to date, the fluxgate magnetic sensor with flip chip configuration offers advances in both device functionality and fabrication simplicity. More importantly, the novel design can be further extended to a silicon-based micro-fluxgate chip manufactured by emerging CMOS-MEMS technologies, thus enriching its potential range of applications in modern engineering and the consumer electronics market.

## 1. Introduction

Fluxgate magnetometers are a class of sensitive sensors used to measure dc or low-frequency magnetic field vectors. Conventional fluxgate sensors typically feature low noise, high sensitivity and excellent accuracy [1], however, they still suffer from the bulky volume imposed by the use of wire-wound coils, high power consumption and low integration capacity when compared to other competitive magnetometers such as magnetoimpedance (MI) sensors and magnetoresistance sensors, including anisotropic-magnetoresistance (AMR), giant-magnetoresistance (GMR), and tunneling magnetoresistance (TMR) devices [2]. Fluxgate magnetometers are promising in applications for military detection, inertial navigation and bio-medical identification [3,4]. Due to the trend of system miniaturization, the development of miniature fluxgate sensors compatible with complementary metal-oxide-semiconductor (CMOS) and micro-electromechanical systems (MEMS) technologies has been emerging in recent years.

A number of research results have reported that micro-fluxgate sensors of several millimeters in size can achieve field noise levels of 70 nT/Hz^1/2^ at 1 Hz [5], 5 nT/Hz^1/2^ at 10 Hz [6], and the minimum power consumption can be less than 10 mW per axis [5,7]. To date potential applications of micro-fluxgates including digital navigation in mobile devices [5], motion tracking [2], thoracoscopic surgery [8], and non-destructive testing [9] have been considerably developed. For compass applications, the dc detectivity should be less than 100 nT with negligible hysteresis. Magnetic field sensors based on MEMS technology, *i.e.*, resonant MEMS sensors [10], can avoid the output hysteresis due to ferromagnetic materials. However, with dimensions of less than 3 mm, their typical detectivity is greater than 100 nT [10] and hence they are not suitable for compass application in mobile devices. In order to meet the requirement of low hysteresis, various field modulation or reset techniques must be applied for those sensors. For magnetoresistance sensors, an additional modulation or reset coil is necessary to suppress the output hysteresis [11–13]. For MI sensors, an additional field or current biasing is required to achieve a bipolar output and reduce the dc hysteresis [14]. However, the output obviously drifts after MI sensors are exposed to a large magnetic field, and this effect cannot be removed completely with the additional biasing [2]. On the contrary, a micro-fluxgate can be driven by ac modulation fields and exhibits a low-hysteresis bipolar output without dc bias source, making it a perfect miniature magnetic field sensor. Regarding the characterization of fluxgate magnetometers, previous studies concluded that the sensing voltage of a fluxgate magnetometer can be fully characterized and thus device sensitivity enhanced by employing the second harmonic or multiple harmonic frequency analyses referred to the excitation signal [1,6,7]. Nevertheless, some manufacturing limitations brought on by micro-fabrication processes have made it necessary to compromise on the performance of micro-fluxgate sensors. In addition to the magnetic core material and its geometry, the design on both excitation and pick-up coils is one of the major factors that dominate the performance of a miniature fluxgate.

Coil configurations in fluxgate sensors can be categorized into planar [15–17] or three-dimensional (3-D) designs [18–20]. The 3-D coils used in traditional wire-wound fluxgate sensors are considered to possess high responsivity and low noise, yet, they still have bulky volume and poor compatibility with interfacing circuits, which are substantial disadvantages for the primary requirements of a compact product. For the planar design, the in-plane spiral coils can be readily fabricated by using the standard CMOS or MEMS processes. However, limitations are imposed by either weak excitation fields generated by the planar coils, or much more complicated MEMS fabrication processes, making it challenging to carry out further improvement of micro-fluxgate devices. To avoid such disadvantages, a CMOS dual-core micro-fluxgate featuring planar pick-up coils and 3D excitation coils, which are implemented by using standard CMOS process and connecting the interconnection metal with bonding wires, was previously proposed and characterized [21]. Moreover, a recent study on the enhanced responsivity and the field noise of similar CMOS micro-fluxgate sensors has been completed, which demonstrated the maximum responsivity of 8.1 V/T while excited at 110 kHz and the minimum field noise of 2.6 nT/Hz^1/2^ at 1 Hz [22]. This class of wire-bonded micro-fluxgate sensors can be considered to offer a good trade-off between device performance and production considerations.

To further develop the micro-fluxgate structure, we present herein a novel design of a Vacquier-Foerster type fluxgate magnetometer featuring 3D excitation coils and double layers of planar pick-up coils by using the “flip chip” concept, which is successfully employed in the current semiconductor packaging sector. The proposed device results in the following two improvements: more protective solenoid-like excitation coils to provide sufficient magnetic flux, and double allocation of pick-up coils to enhance its sensitivity. Both excitation and pick-up coils can be easily implemented by standard CMOS or print circuit board (PCB) fabrication processes. The excitation coils are easily formed by flipping one identical chip or substrate onto the other one, and then soldering them together. In this article, the miniature fluxgate magnetometer is prototyped with two planar PCBs, characterized by simulations and experiments and then compared with traditionally wire-wound fluxgates. In addition, comprehensive characterizations of the magnetic sensor's features such as responsivity modulation, low-frequency field detection and noise spectra are also analyzed and discussed.

## 2. Design and Analysis of the Fluxgate Sensor

### 2.1. Fluxgate Sensor Using the Flip-Chip Concept

**Figure 1.**
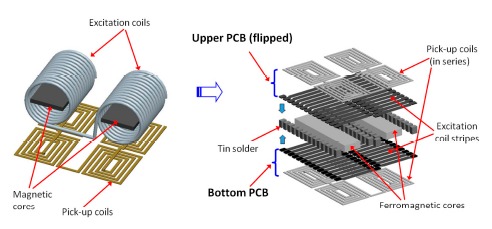
Conceptual schematic of the structure conversion from a wire-wound fluxgate to a “flip chip” PCB fluxgate device.

The “flip chip” or “flip substrate” design for the proposed PCB fluxgate sensor is mainly configured by two 3D excitation coils formed by solder bumps or balls; and four planar pick-up coils fabricated on a PCB substrate via lithographic methods. The simpler fabrication steps and process flow are quite different from those of recently proposed 3-D fluxgate sensors manufactured in more complicated steps [18–20]. Two separate ferromagnetic cores are placed and fixed onto the central positions of excitation coils in the bottom PCB substrate. The Metglas™ 2714A ferromagnetic material (Metglas, Conway, SC, USA) is employed as core and features a low saturation magnetic flux of 10 μT. The upper PCB stubstrate is then flipped; aligned side to side; and bonded to the bottom PCB by tin soldering to construct the 3D excitation coils. The perspective structure of a wire-wound fluxgate transformed to a “flip chip” fluxgate sensor is shown in Figure 1.

It is noted that the layout of the coil stripes on the upper and bottom PCBs is identical but asymmetric. To reduce the excitation signal interference in the planar pick-up coils; the excitation coils are wound in reverse around the magnetic cores so that the magnetic fields in the cores are in opposite directions and the induced voltage can be annulled. Four spiral pick-up coils are connected in series so that the total number of turns is 28 and the total resistance was measured as 3 kΩ at room temperature.

**Figure 2.**
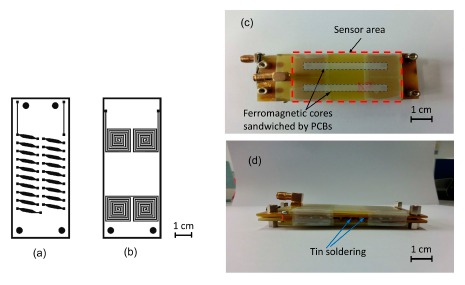
(**a**) Cu patterns of the excitation coil on PCB substrate (front side); (**b**) Cu patterns of the pick-up coil on PCB substrate (back side); (**c**) top view and (**d**) side view of the completed “flip-chip” fluxgate sensor.

The line width of the pick-up coils is 0.5 mm with a gap of 1 mm. The planar pick-up coils are lithographically defined on the backside of each PCB substrate and formed by a dip-etching process. The thick film Cu stripes of the excitation coil configure with stripe width of 2 mm and thickness of 3.5 μm. All Cu stripes of the bottom and upper PCB substrate constitute two solenoid-like excitation coils wrapping the two magnetic cores measured as 40 mm × 3 mm. The total number of turns for two 3-D excitation coils in series is 20 and the total resistance is found to be 2.6 Ω. Therefore, through the “flip substrate” technique, two sets of 3D excitation coils and four pairs of pick-up coils are completed straightforwardly on two PCBs. The main components and the final assembly of the “flip chip” fluxgate sensor are shown schematically in Figure 2.

### 2.2. Theoretical Analysis of a Vacquier-Foerster Fluxgate

A dual-core fluxgate consists of excitation and pick-up coils and two parallel rods of ferromagnetic cores. Assuming that the permeability of the core material is sufficiently high, the induced voltage in the individual pick-up coil can be calculated by using the Faraday's law of induction [3]:

(1) $$V_{i}(t)=-\frac{d\Phi_{i}}{dt}=-NA\frac{dB_{i}\left(t\right)}{dt}$$

where the subscripts 1 or 2 denote the first or second sets of pick-up coils, *N* is the number of turns for each set of pick-up coils, *A* is the cross-section area of each ferromagnetic core, and *B_i_*(*t*) is the magnetic flux density along the longitudal direction of the ferromagnetic core in the *i*-th ferromagnetic core. By taking demagnetization factor *D* into account, the following terms can be defined as [3]:

(2) $$H_{d}=\frac{H_{e}}{1+D\mu_{d}}\;\text{and}\;\mu_{d}=\frac{dB_{i}(t)}{dH_{d}}$$

where *H_e_* is the external dc fields, *H_d_* is the modulated external dc fields by demagnetization factor *D*, and μ*_d_* is the time-dependent permeability under ac excitation. The permeability μ*_d_* is also equivalent to the slop of the *B-H* curve of the core material, and thus the higher it is, the greater the induced voltage exhibits. By substituting Equation (2) into Equation (1), the total output voltage between the two sets of pick-up coils can be expressed as Δ*V* = *V_1_* − *V_2_*, and finally, the quantity of totally induced voltage *V*(*t*) can be approximated by the following equation [3]:

(3) $$V(t)=2V_{i}(t)=-\frac{d}{dt}\left(H_{e}\frac{\mu_{d}}{1+D\mu_{d}}\right)2NA\approx -\frac{2NAH_{e}}{{\left(1+D\mu_{d}\right)}^{2}}\frac{d\mu_{d}}{dt}$$

Equation (3) states that the induced voltage from all pick-up coils is proportional to the external dc fields *H_e_* and the time derivative of permeability μ*_d_*. Also, it can be seen that demagnetization factor *D* is explicit in determining the output voltage. According to previous derivations, the demagnetization factor is approximately related to the ratio of core length *L* and core diameter *d* as the follows [3]:

(4) $$\begin{array}{cc} D\approx {\left(\frac{d}{L}\right)}^{2}\left[2.01\times \log\left(\frac{L}{d}\right)-0.46\right], & L/d\geq 10 \end{array}$$

Since the cross section of the dual-core device is as double as that of the single-core one, the demagnetization factor is estimated less than the two-fold value of itself. More importantly, it is implied by Equation (4) that a narrower and longer magnetic core holding a smaller *d/L* ratio can significantly reduce the demagnetization effect and thus exhibit an increase of the induced voltage in Equation (3). As a result, in addition to high-permeability materials, the dimensional figure of the magnetic core in consideration of decreased demagnetization effect is also critical to improve device responsivity and power consumption for excitation.

### 2.3. Electromagnetic Simulation and Analysis for Sensor Excitation

To compare the effectiveness of the proposed fluxgate with the traditional wire-wound one, we employed the Ansoft Maxwell^©^ electromagnetic software to analyze the distributions of magnetic flux density in the magnetic cores for sensor excitation by using separate models, *i.e.*, traditionally wire-wound and the as-proposed “flip chip” devices. To simplify design diversity, it is noted that, despite the distinct geometric designs of all excitation coils, key design factors such as: (1) the number of wire turns; (2) metal wire gap; (3) cross-sectional area of the metal wire; (4) coil resistance; (5) coil inductance and (6) dimensions of the ferromagnetic core, are chosen to be fully corresponding for each simulation. Therefore, assuming uniform excitation currents through the coils, the following simulation results facilitate the approximate solutions of electromagnetic excitation via the excitation coils and the magnetization phenomena in the magnetic cores.

**Figure 3.**
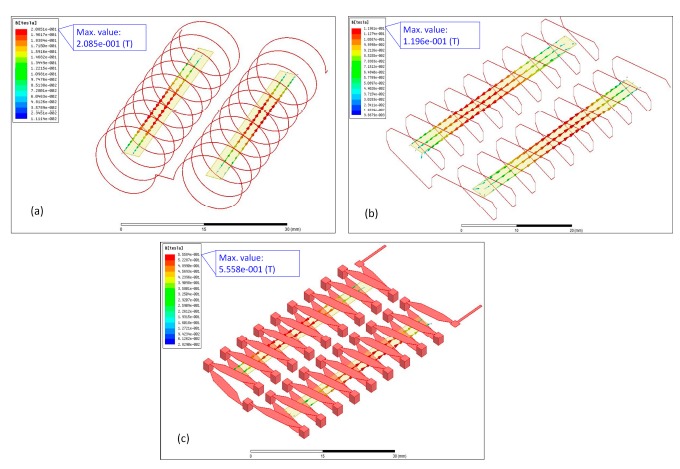
3-D modeling and simulation results of (**a**) cylindrical, wire-wound excitation coils; (**b**) rectangular, wire-wound excitation coils; and (**c**) rectangular, thick-film excitation coils by “flip chip” concept.

3-D solid models for different designs of excitation coils, as shown in Figure 3a–c, were accordingly constructed and analyzed by the FEA simulator. As the function of ac excitation was still unavailable in Ansoft Maxwell^©^ software, a dc excitation mode was adopted instead. Utilizing a 100-mA excitation current and the *B-H* data of Metglas™ 2714A material, it was found that all geometric designs of the excitation coils were able to perform the minimum saturation requirement of induced flux density (>10 μT) through both ends of a core, and among them the proposed “flip chip” design exhibited a maximum value of 0.56 T in the central section of the core. Consequently, it can be inferred that the positive result is attributable to the uniform distribution of magnetic flux by the excitation coils constructed on the PCBs. From the manufacturing point of view, using photo-lithographic and automated soldering operation to produce thick-film excitation coil patterns is considered more batch-productive and cost-effective in comparison with the traditional wiring-process.

## 3. Device Characterizations and Discussions

**Figure 4.**
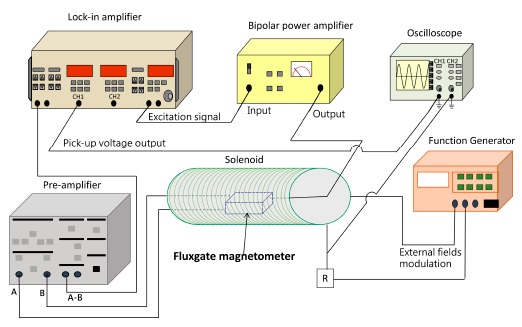
The systematic illustration for characteristic measurement of the fluxgate magnetometer.

To evaluate the miniature fluxgate sensor, one is able to employ an instrumentation system to measure its electromagnetic characteristics. The instrumentation system includes a lock-in amplifier, capable of the second-harmonic detection, to characterize the micro-fluxgate sensor by measuring the voltage output of pick-up coils and generating external (measured) magnetic fields from a modulated solenoid that completely enclose the fluxgate sensor. The systematic diagram for characteristic measurement of the fluxgate is depicted in Figure 4. The instrumentation of the measurement system comprises a lock-in amplifier (SR830, Stanford Research Systems, Sunnyvale, CA, USA), a signal pre-amplifier (SR560, Stanford Research Systems, Sunnyvale, CA, USA), a function generator, a bipolar power amplifier (BA4825, NF Corp., Yokohama, Japan), and a digital-storage oscilloscope. The second harmonic signals sensed by the planar pick-up coils are detected by the lock-in amplifier with a phase reference provided by the function generator. Thus, the sensitivity (or responsivity) of the magnetic sensor, also referred to as the field-to-voltage transfer coefficient *(dV/dB)*, can be obtained by monitoring the pick-up output from the lock-in amplifier and the magnetic fields through a digital oscilloscope.

### 3.1. Responsivity Modulation of the Miniature Fluxgate

**Figure 5.**
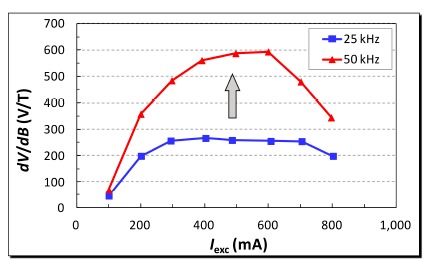
Responsivity of the proposed fluxgate sensor *vs.* the excitation current (*I_exc_*) at various excitation frequencies.

Amplitude and frequency of the excitation current are two major electrical parameters governing the sensor's responsivity and field noise. Therefore, responsivity and field noise can be optimized by tuning operation conditions with these parameters. The responsivity of our fluxgate sensor as a function of the excitation current (*I_exc_*) at various excitation frequencies are investigated and the results given in Figure 5. The responsivity (*dV/dB*) is obtained by detecting their corresponding second harmonics with respect to the frequency of the excitation current. It can be seen that the responsivity, at a lower excitation frequency of 25 kHz, increased to a maximum of 266 V/T at a stable rate up to 300 mA, led to a saturated level till 700 mA then, and finally made a descent down to 199 V/T at the current limit of 800 mA. Comparatively, at a higher excitation frequency of 50 kHz, the responsivity abruptly reached to a maximum of 593 V/T without a saturated phenomenon within 600 mA, and had a direct fall to 344 V/T beyond that point. The *dV/dB* values are generally ascended with rising excitation frequencies with regard to excitation current. These resultant facts were not unusual as our previous study [22] had indicated that the excitation current, when the excitation voltage is fixed, may reduce linearly with respect to the increasing excitation frequency, and it thus increases the responsivity while the excitation frequency goes up, as shown in Figure 5. The reduction in excitation current may be attributed to the increased impedance of the excitation coil at high excitation frequency. The resistance and inductance of the excitation coil were found to be 2.6 Ù and 36 μH at both 25 kHz and 50 kHz. However, the impedance change of such a series L-R circuit is only 0.1% from 10 to 100 kHz, which implies the increase of impedance may also result from the skin effect rather than the inductance variation of the excitation coil. When a 600 mA and a 400 mA excitation currents were applied to drive the sensor under the maximum field-to-voltage transfer at 25 kHz and 50 kHz, and a zero phase shift between the excitation voltage and current waves was arranged, the average power consumption of the excitation coil were estimated as 468 mW and 208 mW, respectively.

Another potential factor that may affect the *dV/dB* ratio is the stray capacitance between the bottom excitation coil and the pick-up coils, resulting in a leakage current at a high frequency. Nevertheless, it was found that the stray capacitance is less than 160 pF, corresponding to a minimum impedance of 3.6 kÙ at 1 MHz. As the minimum impedance is much greater than the coil resistance, the leakage current is negligible for the frequency applied below 1 MHz, which implies that the capacitively coupling effect on the sensor responsivity can be excluded.

### 3.2. Harmonic Characterization and Low-Frequency Field Detections

**Figure 6.**
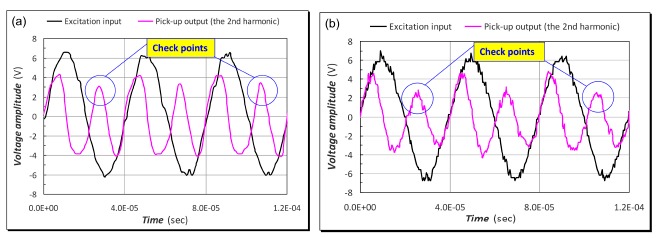
Comparison of the second harmonic waves measured by (**a**) the “flip chip” fluxgate and (**b**) the wire-wound fluxgate under dc earth fields without magnetic shielding. Note that the former exhibits a more noise-resistant effect than the latter by examining the signals in these check points.

Based on the second harmonic frequency principles presented in [1,6,7], the resultant time traces of output voltage of the pick-up coils with a sinusoidal excitation under dc earth fields is shown in Figure 6. It is noted that the fundamental frequency of the induced voltage is the second harmonic of excitation when the *B-H* curves of the two magnetic cores are identical. The induced voltage is found non-sinusoidal due to many high-order even harmonics of excitation. When the external fields is small, the peak amplitude of *V*(*t*) is approximately proportional to *H_e_*, consistent with the prediction of Equation (3). To compare the characteristics of the “flip chip” and wire-wound designs, a wire-wound fluxgate sensor structure was implemented using the equivalent dimensions as described in the previous Section 2.1. By taking a careful and close look into Figure 6a,b, one can see that both of them demonstrated quite similar voltage outputs of the second harmonics with respect to the excitation signals, and the only disparity between the induced voltage reactions was those significant ripples found in the wire-wound case, which further indicates that our “flip-chip” PCB fluxgate sensor may provide a more noise-resistant and stable structure than the traditional devices for weak magnetic field detection.

**Figure 7.**
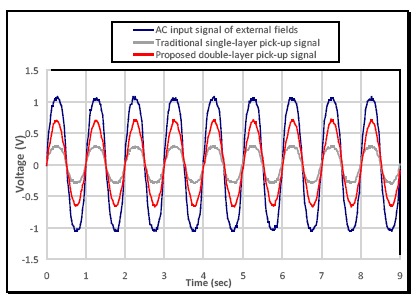
The output voltage of pick-up coils under 1-Hz external fields in terms of single-layer and double-layer layout design of pick-up coils.

**Figure 8.**
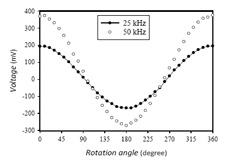
The perfect detection of earth fields as an e-compass at 25 kHz and 50 kHz excitation frequencies using our proposed fluxgate magnetometer.

Moreover, the characterization of the PCB fluxgate magnetometer under low frequency (1 Hz) external magnetic fields driven by a sinusoidal input signal through a solenoid was carried out. The fluxgate magnetometer was placed in the solenoid to detect the homogeneous magnetic fields as single-axis application, and was operated with an excitation current of 600 mA at 50 kHz. Based on the experimental results, as shown in Figure 7, the proposed PCB sensor is proven to allow real-time ac field detection at low frequency of 1 Hz and demonstrate precise voltage fluctuation synchronously with the external fields. More importantly, the double-layer sensing arrangement (see the red curve) connecting all the series pick-up coils both on the upper and bottom PCBs exhibits two-fold voltage amplitude greater than that of the single-layer sensing structure (see the grey curve) which simply exploits one sensor substrate in traditional design. Furthermore, the sensor was demonstrated as an electronic compass by detecting earth fields in our laboratory environment under various excitation frequencies and favorable measurement results obtained (see Figure 8). These facts, again, can justify the core benefits of the miniature fluxgate by using the “flip chip” design proposed in this article.

Also, by observing the *V-B* diagram near the zero-magnetism point under the second harmonic, one is able to evaluate the linearity of the sensor. For example, the *V-B* diagrams with respect to in-phase and quadrature-phase outputs under the second harmonic frequencies (50 kHz and 100 kHz, not shown here) can explicate the liner detection range of magnetic fields. For instance, it was revealed that the maximum responsivity (∼266 V/T), with an excitation current of 300 mA and an excitation frequency of 25 kHz, can bring about a satisfactory linearity range from −10 G to 10 G, which is considered fully eligible for electronic compasses or advanced bio-medical field detection.

### 3.3. Noise Analysis

**Figure 9.**
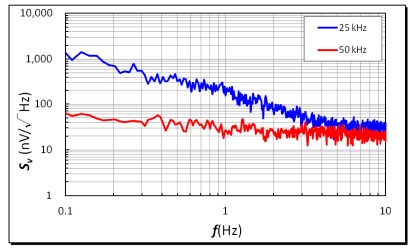
Voltage noise spectral density of the second harmonics under different excitation frequencies.

**Figure 10.**
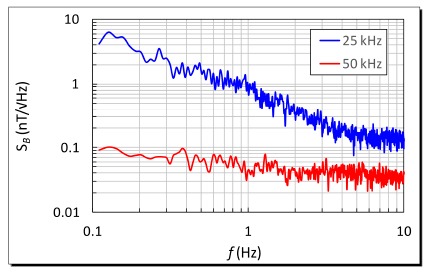
Field noise spectral density of the second harmonics under different excitation frequencies.

Noise analyses of the micro-fluxgate sensor in a shielded environment were investigated and this was considered essential to evaluate the operation feasibility of a miniature magnetometer. It was noted that the voltage noise is generated from electromagnetic components, circuits or ambient environment. The experiments were carried out with an excitation current of 600 mA at 25 kHz and 50 kHz. As depicted in Figure 9, the measurements of voltage noise density at 1 Hz were found to be 202 nV/Hz^1/2^ for a 25 kHz excitation and 32 nV/Hz^1/2^ for a 50 kHz excitation, respectively. In addition, the field noise spectral density of the fluxgate sensor is defined as the ratio of the voltage noise density to its corresponding responsivity. Typically, field noise becomes low when sensor's responsivity is high. Also, it is shown that the voltage noise density may descend rapidly when excitation frequency doubles up, which significantly help reduce field noise spectrum. Thus, the analytic results at 1 Hz indicated that the minimum field noise spectra of 0.79 nT/Hz^1/2^ for a 25 kHz excitation and 0.05 nT/Hz^1/2^ for a 50 kHz excitation were successfully obtained, as shown in Figure 10. Within the low frequency range (0.1∼10 Hz), it was demonstrated that both voltage and field noise response do not exhibit a significant increase in low frequency band.

Further, in comparison with the previously published fluxgate sensors such as MEMS-based micro-fluxgate devices in References [16,18–23], PCB-based fluxgate devices [17,24,25] are normally considered superior to the MEMS micro-fluxgate devices by 1∼2 orders both in the performance of responsivity and field noise due to their larger sensor dimensions.

Apparently, the responsivity of our novel device, compared to that of planar PCB devices [17,24,25], is highly enhanced owing to the stronger excitation field resulting from the “flip chip” structure of the excitation coils, higher permeability of the magnetic cores and double-layer layout of the pick-up coils. As a result, the low field noise of our device reaching a sub-nT/Hz^1/2^ level can be attributed to the extraordinary design of the 3-D flip-chip structure made of two planar PCBs, leading to the elevated responsivity caused by a higher driving frequency (∼50 kHz) or the decreased voltage noise at lower excitation frequency (∼100 Hz). Also, the double-layer pick-up coils enclosing the magnetic cores may provide good coupling to reduce noise. In addition, a design of CMOS low-noise transducing circuits can be employed to decrease the field noise level of the miniature fluxgate system and thus result in the improved detectivity.

### 3.4. Future Work on Flip-Chip Micro-Fluxgate Sensors

**Figure 11.**
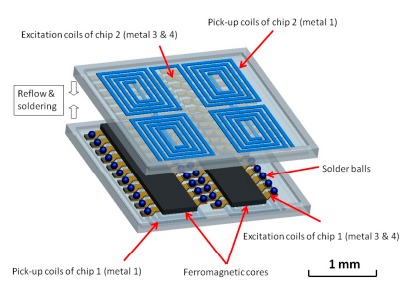
Schematic of the proposed flip-chip micro-fluxgate sensor.

According to the successful results described above, it is highly promising to extend the PCB scale to a micro-silicon chip level and predict the advantages beneficial from the proposed flip-chip concept. First, the device volume and power consumption of the CMOS micro-fluxgate are definitely shrunken to several tens of milliwatts or even lower. Further, compared with the previous wire-bound micro-fluxgate [21,22], the flip-chip micro-fluxgate sensor is able to provide a more homogeneous excitation field and physical protection for the magnetic cores. Besides, and more importantly, the CMOS transducing/driving circuits can be fully integrated with the sensor implemented on a single chip. These benefits will undoubtedly advance the development of state-of-the-art fluxgate magnetometers. However, some critical concerns such as decreased responsivity, elevated noise spectral density and integration requirements for CMOS technology will require more efforts to be clarified. The CMOS micro-fluxgate chip was manufactured by a TSMC 0.35-μm 2P4M mixed-signal CMOS process. The excitation coils are patterned by CMOS process and made of two levels of metal (metal 3 and metal 4 layers) while two pairs of planar pick-up coils (metal 1 layer) are allocated beneath each single ferromagnetic core. The real flip-chip process for chip combination can be realized by using 100 μm-diameter tin solder balls and specific reflow steps. Device assembly and characterization of the CMOS-MEMS micro-fluxgate devices are currently under way and prospective results anticipated. The proposed schematic of the micro-fluxgate magnetometer is revealed in Figure 11.

## 4. Conclusions

A new design and manufacturing strategy for improving the device sensitivity and reducing the field noise of a miniature fluxgate magnetometer implemented by the “flip chip” concept is described for the first time. Such a flipping design results in a straightforward fabrication method for the miniature fluxgate sensor built on PCBs. Besides, characteristic investigations of the magnetic sensor, including responsivity modulation, low-frequency field detection and noise spectral density, are also provided and discussed. With the double-layer installation of planar pick-up coils around the 3-D excitation coils, a maximum responsivity of 593 V/T is successfully obtained when excited at 50 kHz; the minimum magnetic field noise is 0.05 nT/Hz^1/2^ at 1 Hz under the same excitation. Moreover, the dynamic behavior of the proposed sensor that detects a very low frequency (1 Hz) magnetic fields and dc earth fields, and two-fold magnitude of the sensing voltage (responsivity) from the pick-up coils are evidently demonstrated. The PCB-level verifications for the reported miniature fluxgate sensor can provide outstanding pre-work to develop a real flip-chip micro-fluxgate magnetometer based on CMOS-MEMS technologies.
